# Supplementary material for: A multi-scale digital twin for adiposity-driven insulin resistance in humans: diet and drug effects
Source: Diabetol Metab Syndr. 2023 Dec 4;15:250. doi: 10.1186/s13098-023-01223-6 (PMC10694923; doi:10.1186/s13098-023-01223-6)
Supplement: Supplementary file 1 — Additional file 1: Table S1. Parameters of the cell level that were not changed. The values used were the same as in (Brännmark et al. 2013) for both the Topiramate Study and the Fast-food study. Table S2. Parameters of the cell level that were changed. Fitted so that IRtot and GLUT4 are at 100 % in steady state. The same values were used for both the Topiramate Study and the Fast-food study. Table S3. Parameters on the organ/tissue level that were set to different values for the different studies. Table S4. Parameters of the organ/tissue level that were kept at the same values for both the Fast-food study and Topiramate study data. The values come from (Herrgårdh et al. 2021). Table S5. Parameters of the whole-body level that were set to different values for the different studies. Table S6. Parameters of the whole-body level that were kept at the same values for both the Fast-food study and Topiramate study data. Table S7. Parameters of the topiramate model used on the Topiramate study data. The values come from (Girgis et al. 2010). Table S8. Initial values of cell level model. For the Topiramate study, these values were only used for the predictions (Fig. 5 E–G). These values were obtained through steady state simulation. Table S9. Initial values of organ/tissue level model. For the Topiramate study, these values were only used for the predictions (Fig. 5 E–G). Table S10. Initial values of whole-body level model used in model training and validating. Table S11. Initial values of topiramate model used in model training and validating for Topiramate study. Figure S1. No uncertainties that made the cut-off were found for the weight data. Figure S2. Results of validation on fat and lean mass. Figure S3. Results of validation on fasting glucose and insulin [file 13098_2023_1223_MOESM1_ESM.pdf]

Supplementary Material

A multi-scale digital twin for adiposity-driven insulin resistance in humans: diet and drug effects

All the ODEs for the final model:

$$\frac{d}{dt}(IRm) = -v1a - v1basal + v1r + v1g + vIR \tag{1}$$

$$\frac{d}{dt}(IRp) = v1basal + v1c - v1d - v1g \tag{2}$$

$$\frac{d}{dt}(IRins) = v1a - v1c \tag{3}$$

$$\frac{d}{dt}(IRip) = v1d - v1e \tag{4}$$

$$\frac{d}{dt}(IRi) = v1e - v1r - vIRdeg \tag{5}$$

$$\frac{d}{dt}(IRS1) = v2b + v2g - v2a - v2basal \tag{6}$$

$$\frac{d}{dt}(IRS1p) = v2a + v2d - v2b - v2c \tag{7}$$

$$\frac{d}{dt}(IRS1p307) = v2c - v2d - v2f \tag{8}$$

$$\frac{d}{dt}(IRS1307) = v2basal + v2f - v2g \tag{9}$$

$$\frac{d}{dt}(X) = v3b - v3a \tag{10}$$

$$\frac{d}{dt}(Xp) = v3a - v3b \tag{11}$$

$$\frac{d}{dt}(PKB) = -v4a + v4b + v4h \tag{12}$$

$$\frac{d}{dt}(PKB308p) = v4a - v4b - v4c \tag{13}$$

$$\frac{d}{dt}(PKB473p) = -v4e + v4f - v4h \tag{14}$$

$$\frac{d}{dt}(PKB308p473p) = v4c + v4e - v4f \tag{15}$$

$$\frac{d}{dt}(mTORC1) = v5b - v5a \tag{16}$$

$$\frac{d}{dt}(mTORC1a) = v5a - v5b \tag{17}$$

$$\frac{d}{dt}(mTORC2) = -v5c + v5d \tag{18}$$

$$\frac{d}{dt}(mTORC2a) = v5c - v5d \tag{19}$$

$$\frac{d}{dt}(AS160) = v6b1 - v6f1 \tag{20}$$

$$\frac{d}{dt}(AS160p) = v6f1 - v6b1 \tag{21}$$

$$\frac{d}{dt}(GLUT4m) = (v7f - v7b - vGLUTdeg) \tag{22}$$

$$\frac{d}{dt}(GLUT4) = -v7f + v7b + vGLUT \tag{23}$$

$$\frac{d}{dt}(S6K) = v9b1 - v9f1 \tag{24}$$

$$\frac{d}{dt}(S6Kp) = v9f1 - v9b1 \tag{25}$$

$$\frac{d}{dt}(S6) = v9b2 - v9f2 \tag{26}$$

$$\frac{d}{dt}(S6p) = v9f2 - v9b2 \tag{27}$$

$$\frac{d}{dt}(G_p) = EGP + Ra - E - U_{ii} - k_1 \cdot G_p + k_2 \cdot G_t \tag{28}$$

$$\frac{d}{dt}(G_t) = -U_{id} + k_1 \cdot G_p - k_2 \cdot G_t - U_{idl} \tag{29}$$

$$\frac{d}{dt}(I_l) = (-m_1 \cdot I_l) - m_3 \cdot I_l + m_2 \cdot I_p + S \tag{30}$$

$$\frac{d}{dt}(I_p) = (-m_2 \cdot I_p) - m_4 \cdot I_p + m_1 \cdot I_l \tag{31}$$

$$\frac{d}{dt}(Q_{sto1}) = -k_{gri} \cdot Q_{sto1} \tag{32}$$

$$\frac{d}{dt}(Q_{sto2}) = (-k_{empt} \cdot Q_{sto2}) + k_{gri} \cdot Q_{sto1} \tag{33}$$

$$\frac{d}{dt}(Q_{gut}) = (-k_{abs} \cdot Q_{gut}) + k_{empt} \cdot Q_{sto2} \tag{34}$$

$$\frac{d}{dt}(I_1) = -k_i \cdot (I_1 - I) \tag{35}$$

$$\frac{d}{dt}(I_d) = -k_i \cdot (I_d - I_1) \tag{36}$$

$$\frac{d}{dt}(INS_f) = (-p_{2U} \cdot INS_f) + p_{2U} \cdot (I - I_b) \tag{37}$$

$$\frac{d}{dt}(I_{po}) = ((-gamma \cdot I_{po}) + S_{po}) \cdot f_{IR_{Ins}} \tag{38}$$

$$\frac{d}{dt}(Y) = -alpha \cdot (Y - beta \cdot (G - G_b)) \tag{39}$$

$$\frac{d}{dt}(INS) = V_1 - V_2 \tag{40}$$

$$\frac{d}{dt}(Glu_{in}) = p1 \cdot (V_{in} - V_{out}) - V_{G6P} \tag{41}$$

$$\frac{d}{dt}(G6P) = V_{G6P} - V_{met} \tag{42}$$

$$\frac{d}{dt}(Gly) = \frac{v1}{rhog} \tag{43}$$

$$\frac{d}{dt}(ECF) = v2 \tag{44}$$

$$\frac{d}{dt}(F) = \frac{(1 - v3) \cdot (k1 - v1)}{rhof} \tag{45}$$

$$\frac{d}{dt}(L) = \frac{v3 \cdot (k1 - v1)}{rhol} \tag{46}$$

$$\frac{d}{dt}(AT) = v4 \tag{47}$$

$$\frac{d}{dt}(A) = -Ka \cdot A \tag{48}$$

$$\frac{d}{dt}(C) = Ka \cdot \frac{A}{V} - K23 \cdot C + K23 \cdot y3 - k10 \cdot C \tag{49}$$

$$\frac{d}{dt}(C1) = K32 \cdot C - K32 \cdot C1 \tag{50}$$

All variables of final model:

$$aa = \frac{5/2}{(1-b)/D} \tag{51}$$

$$cc = \frac{5/2}{d/D} \tag{52}$$

$$EGP = (k_{p1} - k_{p2} \cdot G_p - k_{p3} \cdot I_d - k_{p4} \cdot I_{po}) \cdot f_{IR_{EGP}} \tag{53}$$

$$V_{lmax} = V_l + V_{lX} \cdot INS \tag{54}$$

$$V_{mmax} = V_m + V_{mX} \cdot INS \tag{55}$$

$$E = 0 \tag{56}$$

$$S = gamma \cdot I_{po} \tag{57}$$

$$I = \frac{I_p}{V_I} \tag{58}$$

$$G = \frac{G_p}{V_G} \tag{59}$$

$$HE = (-m_5 \cdot S) + m_6 \tag{60}$$

$$m_3 = HE \cdot \frac{m_1}{(1-HE)} \tag{61}$$

$$Q_{sto} = Q_{sto1} + Q_{sto2} \tag{62}$$

$$Ra = f \cdot k_{abs} \cdot \frac{Q_{gut}}{BW} \tag{63}$$

$$k_{empt} = k_{min} + \frac{(k_{max} - k_{min})}{2} \cdot (tanh(aa \cdot (Q_{sto} - b \cdot D)) - tanh(cc \cdot (Q_{sto} - d \cdot D)) + 2) \tag{64}$$

$$bf = (be + kbf \cdot (INS_f + INS_{\text{offset}})) \cdot bradykinin \tag{65}$$

$$bfe_f = (bf - bf_b) \cdot (INS_f - INS_b) \cdot p_{bf} \tag{66}$$

$$S_{po} = Y + K \cdot \frac{(EGP + Ra - E - U_{ii} - k_1 \cdot G_p + k_2 \cdot G_t)}{V_G} + S_b \tag{67}$$

$$INS_{fe} = nC \cdot (k8 \cdot \frac{GLUT4m}{pf} + \frac{GLUT1}{pf} + bfe_f) \tag{68}$$

$$V_{in} = p4 \cdot G_t \cdot INS_{fe} \tag{69}$$

$$V_{out} = p3 \cdot Glu_{in} \tag{70}$$

$$U_{idm} = xLV_{mmax} \cdot \frac{G_t}{(K_m + G_t) f_{IR_{CLGI}}} \tag{71}$$

$$U_{idl} = V_{lmax} \cdot \frac{G_t}{(K_l + G_t) f_{IR_{CLGI}}} \tag{72}$$

$$U_{idf} = xFp_5 \cdot (V_{in} - V_{out}) \tag{73}$$

$$U_{id} = U_{idf} + U_{idm} + U_{idl} \tag{74}$$

$$U = U_{ii} + U_{id} + U_{idl} \tag{75}$$

$$V_2 = k_2 \cdot INS \tag{76}$$

$$V_1 = k_1 \cdot (I - I_b) \tag{77}$$

$$v1a = IRm \cdot k1a \cdot (INS_f + 5) \cdot 1e-3 \tag{78}$$
$$v1basal = k1basal \cdot IRm \tag{79}$$
$$v1c = IRins \cdot k1c \tag{80}$$
$$v1d = IRp \cdot k1d \tag{81}$$
$$v1e = IRip \cdot k1f \cdot Xp \tag{82}$$
$$v1g = IRp \cdot k1g \tag{83}$$
$$v1r = IRi \cdot k1r \tag{84}$$
$$v2a = IRS1 \cdot k2a \cdot IRip \tag{85}$$
$$v2b = IRS1p \cdot k2b \tag{86}$$
$$v2c = IRS1p \cdot k2c \cdot mTORC1a \cdot diabetes \tag{87}$$
$$v2d = IRS1p307 \cdot k2d \tag{88}$$
$$v2f = IRS1p307 \cdot k2f \tag{89}$$
$$v2basal = IRS1 \cdot k2basal \tag{90}$$
$$v2g = IRS1307 \cdot k2g \tag{91}$$
$$v3a = X \cdot k3a \cdot IRS1p \tag{92}$$
$$v3b = Xp \cdot k3b \tag{93}$$
$$v5a = mTORC1 \cdot (k5a1 \cdot PKB308p473p + k5a2 \cdot PKB308p) \tag{94}$$
$$v5b = mTORC1a \cdot k5b \tag{95}$$
$$v5c = mTORC2 \cdot k5c \cdot IRip \tag{96}$$
$$v5d = k5d \cdot mTORC2a \tag{97}$$
$$v4a = k4a \cdot PKB \cdot IRS1p \tag{98}$$
$$v4b = k4b \cdot PKB308p \tag{99}$$
$$v4c = k4c \cdot PKB308p \cdot mTORC2a \tag{100}$$
$$v4e = k4e \cdot PKB473p \cdot IRS1p307 \tag{101}$$
$$v4f = k4f \cdot PKB308p473p \tag{102}$$
$$v4h = k4h \cdot PKB473p \tag{103}$$
$$v6f1 = AS160 \cdot (k6f1 \cdot PKB308p473p + k6f2 \cdot \frac{PKB473p^n6}{(km6^n6 + PKB473p^n6))} \tag{104}$$
$$v6b1 = AS160p \cdot k6b \tag{105}$$
$$v7f = GLUT4 \cdot k7f \cdot AS160p \tag{106}$$
$$v7b = GLUT4m \cdot k7b \tag{107}$$
$$v9f1 = S6K \cdot k9f1 \cdot \frac{mTORC1a^n9}{km9^n9 + mTORC1a^n9} \tag{108}$$
$$v9b1 = S6Kp \cdot k9b1 \tag{109}$$
$$v9f2 = S6 \cdot k9f2 \cdot S6Kp \tag{110}$$
$$v9b2 = S6p \cdot k9b2 \tag{111}$$
$$V_{G6P} = V_{G6P_{max}} \cdot \frac{Glu_{in}}{(k_{gluin} + Glu_{in})} \cdot \frac{1}{(k_{G6P} + G6P)} \tag{112}$$
$$V_{met} = p3 \cdot G6P \tag{113}$$

$$xF = \frac{F}{Finit} \tag{114}$$

$$xL = \frac{L}{Linit} \tag{115}$$

$$f_{IR_{CLGI}} = 1 + (b_{CLGI} \cdot log(xF)) \cdot scale \tag{116}$$

$$f_{IR_{EGP}} = 1 + (b_{EGP} \cdot log(xF)) \cdot scale \tag{117}$$

$$f_{IR_{Ins}} = 1 + (b_{Ins} \cdot log(xF)) \cdot scale \tag{118}$$

$$f_{IR_{ir}} = 1 - b_{IR} \cdot log(xF) \tag{119}$$

$$f_{IR_{glut4}} = 1 - b_{GLUT} \cdot log(xF) \tag{120}$$

$$diabetes = 1 - b_{diabetes} \cdot log(xF) \tag{121}$$

$$CC = 10.4 \cdot \frac{rhol}{rhof} \tag{122}$$

$$p = \frac{CC}{(CC + F)} \tag{123}$$

$$EEinit = PAE \cdot RMRinit \tag{124}$$

$$EIninit = EEinit \tag{125}$$

$$CIninit = fCIn \cdot EIninit \tag{126}$$

$$kg = \frac{CIninit}{Ginit \cdot Ginit} \tag{127}$$

$$dEI_{i}nit = EIrestriction \cdot joules \tag{128}$$

$$IC = 1 - lmax \cdot \frac{C^{h2}}{(C^{h2} + IC_{50}^{h2})} \tag{129}$$

$$EI_{vehicle} = EIninit + dEI_{init} - (dEI_{init} - dEI_{ss} \cdot joules) \cdot \frac{time^{h1}}{(time^{h1} + t_{half}^{h1})} \tag{130}$$

$$EIn = EI_{vehicle} \cdot IC \tag{131}$$

$$CInvalue = fCIn \cdot EI_{vehicle} \tag{132}$$

$$CIn = CIninit \cdot CInvalue \tag{133}$$

$$PAL = PAE \tag{134}$$

$$BW = (F + L + (1 + 2.7) \cdot Gly + ECF) \tag{135}$$

$$TEF = bTEF \cdot (EIn - EIninit) \tag{136}$$

$$KK = EEinit - (gf \cdot Finit + gl \cdot Linit + delta \cdot BWinit) \tag{137}$$

$$EE1 = -BW \cdot delta \cdot rhof \cdot rhol - KK \cdot rhof \cdot rhol - rhof \cdot rhol \cdot TEF - rhof \cdot rhol \cdot AT \tag{138}$$

$$+ etal \cdot p \cdot rhof \cdot CIn + etaf \cdot rhol \cdot CIn - etaf \cdot p \cdot rhol \cdot CIn - etal \cdot p \cdot rhof \cdot EIn \tag{139}$$

$$- etaf \cdot rhol \cdot EIn + etaf \cdot p \cdot rhol \cdot EIn - gf \cdot rhof \cdot rhol \cdot F \tag{140}$$

$$- etal \cdot kg \cdot p \cdot rhof \cdot Gly \cdot Gly - etaf \cdot kg \cdot rhol \cdot Gly \cdot Gly \tag{141}$$

$$+ etaf \cdot kg \cdot p \cdot rhol \cdot Gly \cdot Gly - gl \cdot rhof \cdot rhol \cdot L \tag{142}$$

$$EE = \frac{EE1}{(-etal \cdot p \cdot rhof - etaf \cdot rhol + etaf \cdot p \cdot rhol - rhof \cdot rhol)} \tag{143}$$

$$k1 = EIn - EE \tag{144}$$

$$vIR = kIR \cdot f_{IR_{ir}} \tag{145}$$

$$vIRdeg = kIRdeg \cdot IRi \tag{146}$$

$$vGLUT = kGLUT \cdot f_{IR_{glut4}} \tag{147}$$

$$vGLUTdeg = kGLUTdeg \cdot GLUT4m \tag{148}$$

$$IRtot = IRm + IRins + IRp + IRip + IRi \tag{149}$$

$$GLUT4tot = GLUT4 + GLUT4m \tag{150}$$

Annotation	Value	Annotation	Value
$k1a$	0.633141	$k6f2$	36.9348
$k1basal$	0.0331338	$km6$	30.5424
$k1c$	0.876805	$n6$	2.13707
$k1d$	31.012	$k6b$	65.1841
$k1f$	1839.58	$k7f$	50.9829
$k1g$	1944.11	$k7b$	2285.97
$k1r$	0.547061	$k8$	724.242
$k2a$	3.22728	$glut1$	7042.19
$k2c$	5758.78	$k9f1$	0.12981
$k2basal$	0.0422768	$k9b1$	0.0444092
$k2b$	3424.35	$k9f2$	3.3289
$k2d$	280.753	$k9b2$	30.9967
$k2f$	2.9131	$km9$	5872.68
$k2g$	0.267089	$n9$	0.985466
$k3a$	0.00137731	kbf	0.01
$k3b$	0.0987558	nC	2.1e-06
$k4a$	5790.17	INS_{offset}	7
$k4b$	34.7965	k_{gluin}	2.14
$k4c$	4.45581	$p1$	0.179
$k4e$	42.8395	$p2$	4.48
$k4f$	143.597	$p3$	0.161
$k4h$	0.536145	$p4$	2.63
$k5a1$	1.8423	k_{G6P}	11495
$k5a2$	0.055064	V_{G6Pmax}	410
$k5b$	24.826	$k6f1$	2.65168
$k5d$	1.06013	$k5c$	0.0857515
$km5$	2.64988		

Table S1: Parameters of the cell level that were not changed. The values used were the same as in (Brännmark et al. 2013) for both the Topiramate Study and the Fast-food study.

Annotation	Value
kIR	0.0323845
$kIRdeg$	1
$kGLUT$	62.81266
$kGLUTdeg$	1

Table S2: Parameters of the cell level that were changed. Fitted so that $IRtot$ and $GLUT4$ are at 100 % in steady state. The same values were used for both the Topiramate Study and the Fast-food study.

Annotation	Value used on Fast-food study data (estimated)	Value used on Topiramate study data (Herrgårdh et al. 2021)
V_G	1.98	1.88
k_1	0.0613	0.065
k_2	0.0779	0.079
G_b	86.2	95
V_I	0.0538	0.05
m_1	0.208	0.19
m_2	0.517	0.484
m_4	0.192	0.194
m_5	0.0274	0.0304
m_6	0.5921	0.6471
HE_b	0.660	0.6
I_b	27.5	25
S_b	1.83	1.8
k_{max}	0.0525	0.0558
k_{min}	0.00878	0.008
k_{abs}	0.0624	0.057
k_{gri}	0.0592	0.0558
f	0.919	0.9
b	0.741	0.82
dd	0.0106	0.01
k_{p1}	2.43	2.7
k_{p2}	0.00209	0.0021
k_{p3}	0.00968	0.009
k_{p4}	0.0557	0.0618
k_i	0.00714	0.0079
p_{2U}	0.0316	0.0331
K	2.49	2.3
$alpha$	0.0480	0.05
$beta$	0.101	0.11
$gamma$	0.492	0.5
k_{e10}	0.000458	0.0005
k_{e2}	345	339
D	67600 / 68110	101000 / 94759

Table S3: Parameters on the organ/tissue level that were set to different values for the different studies. For the Fast-food study, the parameters were estimated on initial values of glucose and insulin. For the Topiramate study, the values from (Herrgårdh et al. 2021) were used. The meal, D (set to the same as the initial value of Q_{sto}), was set to 1 g/kg of body weight. The two values for D are for the meals simulated in the before/after scenarios respectively.

Annotation	Value
U_{ii}	0.831
$k2$	0.0429
$k1$	0.0476
V_m	0.881
V_{mx}	0.0409
K_m	476
V_l	2.00
V_{lx}	0.0439
K_l	355
bf_b	0
$bradykinin$	1
be	3
INS_b	0.8549
p_{bf}	5

Table S4: Parameters of the organ/tissue level that were kept at the same values for both the Fast-food study and Topiramate study data. The values were come from (Herrgårdh et al. 2021).

annotation	Value used on Topiramate study data (Herrgårdh et al. 2021)	Value used on Fast-food study data (estimated)
$dosage$	0 / 64 / 192 / 96	0
$BWinit$	105.4 / 103.1 / 101 / 104.6	67.6
$height$	166.5 / 166.2 / 166.1 / 166.8	175.6917
age	43.6 / 45.1 / 44.7 / 45	27
$RMRinit$	7189504 / 7054940 / 6971848 / 7133704	7102549
$Ginit$	1.85	1.85
$ECFinit$	17.34 / 16.96 / 16.6145 / 17.21	11.12
$Finit$	52.12 / 50.44 / 48.59 / 51.49	12.7
$Linit$	29.09 / 28.86 / 28.95 / 29.06	36.9348
$ATinit$	0.1	0.1
$EIrestriction$	-600	3480
k	0.010161196182943	0
$lmax$	0.161411312363267	0
IC_{50}	0.149118981539457	1
$h2$	1	1
$h1$	0.563148507725789	4.999973082721017
dEI_{ss}	72.848728668834810	1.226464396357887e+03
t_{half}	19.999999526293507	1.424047740519107

Table S5: Parameters of the whole-body level that were set to different values for the different studies. For the Fast-food study, the parameters were estimated on initial values of glucose and insulin. For both studies, the values were either taken from the data ($dosage$, $BWinit$, $height$, age, $Finit$, $Linit$, $EIrestriction$), if not present in data calculated based on the equations in (Hall et al. 2011) and data ($RMRinit$, $Ginit$, $ECFinit$, $Finit$, $Linit$, $ATinit$), or estimated to data from the Topiramate study (k, $lmax$, IC_{50}, and $h2$ to data for dosages 64 and 192, and $h1$, dEI_{ss}, and t_{half} to placebo data). The different values for $dosage$, $BWinit$, $height$, age, $RMRinit$, $ECFinit$, $Finit$, $Linit$, and $EIrestriction$ are the data for the different dosages and their data sets. The meal, D (set to the same as the initial value of Q_{sto}), was set to 1 g/kg of body weight. The two values are for the before/after scenarios respectively. See (Hall et al. 2011) for descriptions of parameters.

Annotation	Value
$joules$	4183
$fCIn$	0.4
PAE	1.5
$rhol$	7.6e6
$rhof$	39.5e6
gf	13000
gl	92000
$etal$	960000
$etaf$	750000
$rhog$	17.6e6
Na	3.22
$epNa$	3000
$epCI$	4000
$deltaNaDiet$	0
$bTEF$	0.1
bAT	0.14
tAT	14
$scale$	2.35
b_{CLGI}	0.605258205407204
b_{EGP}	1.142839561915420
b_{Ins}	0.749598731223457
b_{IR}	1.3696
b_{GLUT}	1.5865
$b_{diabetes}$	0.8962
$delta$	30

Table S6: Parameters of the whole-body level that were kept at the same values for both the Fast-food study and Topiramate study data. The values come from (Hall et al. 2011). See (Hall et al. 2011) for descriptions of parameters.

Annotation	Value
$joules$	4183
CL	1.21
V	4.61
$k10$	0.2625
Ka	0.105
$K23$	0.577
$K32$	0.0586

Table S7: Parameters of the topiramate model used on the Topiramate study data. The values come from (Girgis et al. 2010)

Annotation	Value used on Topiramate study data (Herrgårdh et al. 2021)	Value used on Fast-food study data (estimated)
$IRm(0)$	79.702633787375790	79.702633641196710
$IRp(0)$	0.001616091202093	0.001616091199129
$IRins(0)$	0.287638353419841	0.287638352892073
$IRip(0)$	19.975821355446970	19.975821259258453
$IRi(0)$	0.032384504361388	0.032384504291868
$IRS1(0)$	0.636270043962264	0.636270048741165
$IRS1p(0)$	0.004886920066483	0.004886920079528
$IRS1p307(0)$	8.336225350721554	8.336225373343643
$IRS1307(0)$	91.022683564690510	91.022683812180720
$X(0)$	99.993271954939890	99.993271954921270
$Xp(0)$	0.006815145060111	0.006815145078301
$PKB(0)$	0.607375249849608	0.607375246877010
$PKB308p(0)$	0.055433526447741	0.055433526415003
$PKB473p(0)$	28.457706154130346	28.457706084935324
$PKB308p473p(0)$	70.879375465504100	70.879375485218280
$mTORC1(0)$	15.974509276998015	15.974509273264948
$mTORC1a(0)$	84.025510723002180	84.025510726735000
$mTORC2(0)$	38.229246047154106	38.229246160869980
$mTORC2a(0)$	61.770764952846040	61.770764839130210
$AS160(0)$	24.123630120570360	24.123630120162524
$AS160p(0)$	75.876369879429820	75.876369879837410
$GLUT4m(0)$	62.812699999999055	62.812699999999130
$GLUT4(0)$	37.187401271425344	37.187401271225625
$S6K(0)$	95.802072569195220	95.802072569021830
$S6Kp(0)$	4.197900430804610	4.197900430977912
$S6(0)$	68.925773402622870	68.925773401738680
$S6p(0)$	31.074136597377260	31.074136598261480
$Glu_{in}(0)$	6.219327923337478	5.491579474540069
$G6P(0)$	0.005931193832426	0.005736832943166

Table S8: Initial values of cell level model. For the Topiramate study, these values were only used for the predictions (Fig. 5 E-G). These values were obtained through steady state simulation.

Annotation	Value used on Topiramate study data (Herrgårdh et al. 2021)	Value used on Fast-food study data (estimated)
$G_p(0)$	179.7181945729903	166.8653475365416
$G_t(0)$	134.5262549302064	120.2490760033025
$I_l(0)$	5.683414265848025	5.327358533997125
$I_p(0)$	1.592697213143252	1.566977929053884
$Q_{sto1}(0)$	0 / 101000	0
$Q_{sto2}(0)$	0	0
$Q_{gut}(0)$	0	0
$I_1(0)$	31.853944262865035	29.117931918456380
$I_d(0)$	31.853944262865035	29.117931918456380
$I_{po}(0)$	3.730852556413765	3.335612126317704
$Y(0)$	0.065426278206883	0.187892188500337
$INS_f(0)$	0	1.068266605322548e-19
$INS(0)$	0	1.796308844088321

Table S9: Initial values of organ/tissue level model. For the Topiramate study, these values were only used for the predictions (Fig. 5 E-G). These values were obtained through steady state simulation, except for $Q_{sto1}(0)$ which was set to a meal corresponding to 1 g/kg body weight, or 0 if no meal.

Annotation	Value used on Topiramate study data (Herrgårdh et al. 2021)	Value used on Fast-food study data (estimated)
$Gly(0)$	1.85	1.85
$ECF(0)$	17.34 / 16.96 / 16.6145 / 17.21	11.1202
$F(0)$	52.12 / 50.44 / 48.59 / 51.49	12.7
$L(0)$	29.09 / 28.86 / 28.95 / 29.06	36.9348
$AT(0)$	0.1	0.1

Table S10: Initial values of whole-body level model used in model training and validating. The different values for the Topiramate study correspond to the different dosages 0 / 64 / 192 / 96. For both studies, the values were either taken from the data ($F(0)$, and $L(0)$), or if not present in data calculated based on the equations in (Hall et al. 2011) and data ($RMR(0)$, $Gly(0)$, $ECF(0)$, $F(0)$, $L(0)$, $AT(0)$).

Annotation	Values used for dosage 64 mg/day	Values used for dosage (192 mg/day)	Values used for dosage 96 mg/day (validation)
$A(0)$	16 / 32 / 64	16 / 32 / 64 / 96 / 128 / 192	16 / 32 / 64 / 96
$C(0)$	0	0	0
$C2(0)$	0	0	0

Table S11: Initial values of topiramate model used in model training and validating for Topiramate study. The dosage of topiramate is given as an initial value of the state A, and the different values for each dosage represents the escalation of dosage taken seen in the data in (Bray et al. 2003).

**Full-length name**	**Abbrevation**
Insulin receptor	IR
¡— Insulin receptor substrate 1	IRS1
Protein kinase B	PKB
Endogenous glucose production	EGP
Insulin in the portal vein	Ipo
Ordinary differential equations	ODEs
Food and drug administration	FDA
Fat mass	FM
Fat free mass	FFM
Energy intake	EI
Energy expenditure	EE
Body weight	BW
Phosphorylated PKB	PKB308-p
Phosphorylated IRS1	IRS1-p

Table S12: Abbreviations.

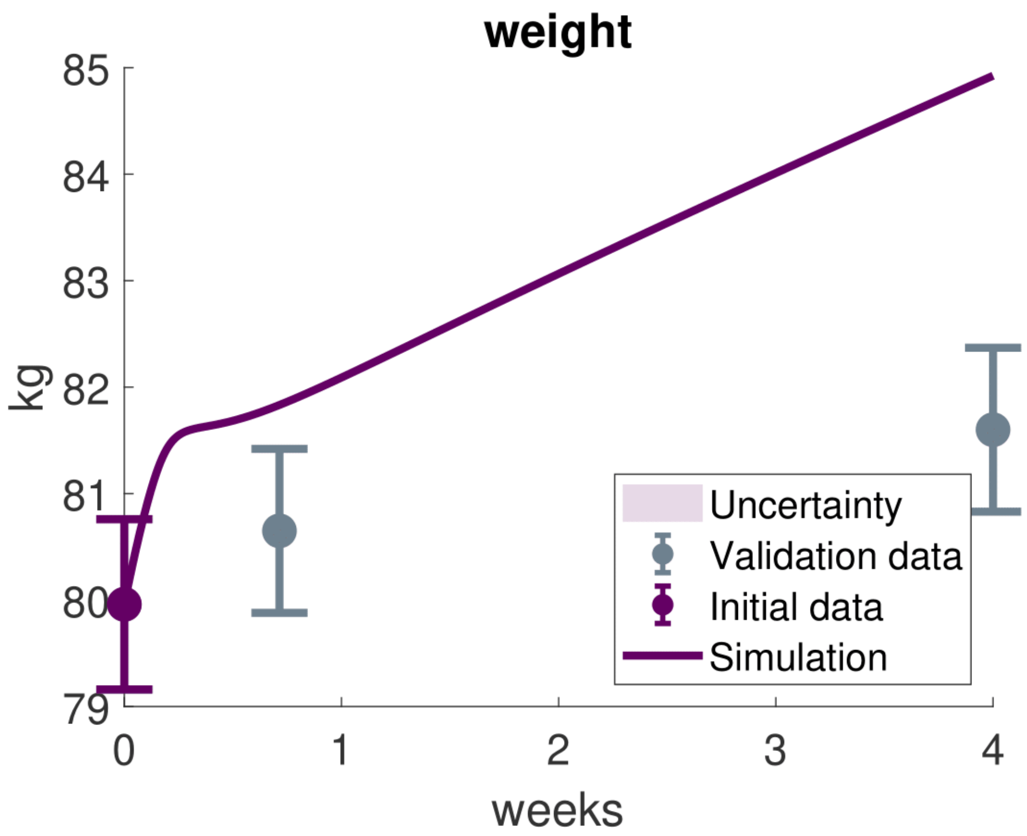

S1: *No uncertainties that made the cut-off were found for the weight data. The simulation shown is using the parameters from the best fit to the Fast food study, but with changed initial weight and energy intake. The simulation clearly overpredicts the data.*

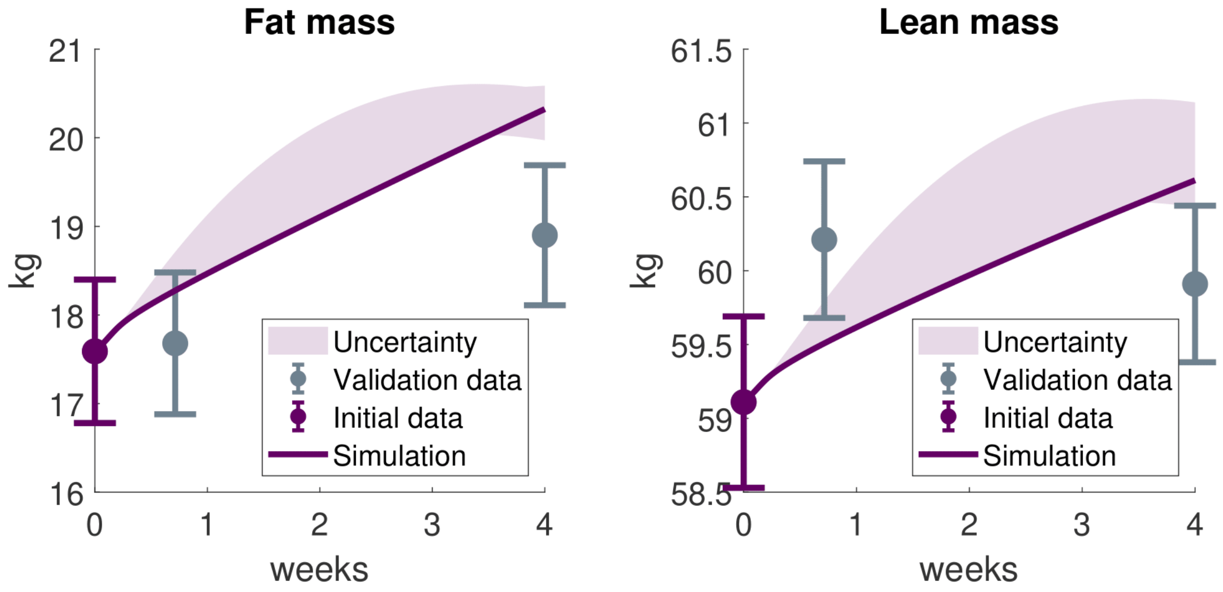

S2: *Results of validation on fat and lean mass.*

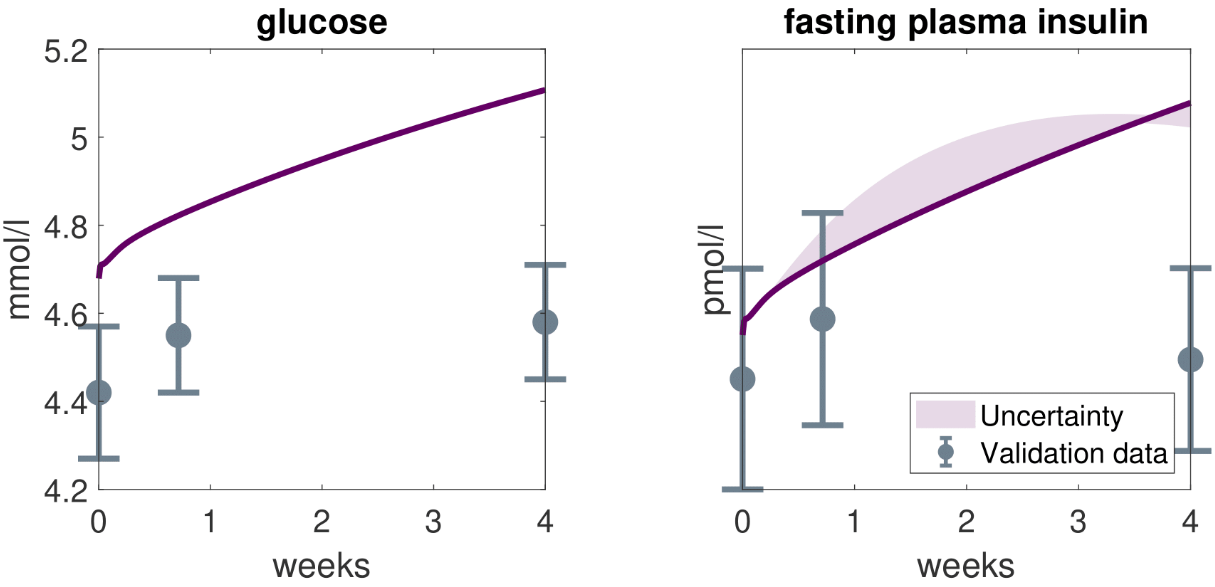

S3: *Results of validation on fasting glucose and insulin.*
